# Differential expression of drug resistance genes in CD146 positive dental pulp derived stem cells and CD146 negative fibroblasts

**DOI:** 10.1002/cre2.297

**Published:** 2020-05-07

**Authors:** Maryam S. Tavangar, Armin Attar, Mahboobeh Razmkhah, Seyed‐Mojtaba Hosseini, Ahmad Hosseini, Ahmad Monabati, Fereshteh Shafiei

**Affiliations:** ^1^ Oral and Dental Disease Research Center, Department of Operative Dentistry School of Dentistry, Shiraz Universityof Medical Sciences Shiraz Iran; ^2^ Department of Cardiovascular Medicine Shiraz University of Medical Sciences Shiraz Iran; ^3^ Shiraz Institute for Cancer Research, School of Medicine Shiraz University of Medical Sciences Shiraz Iran; ^4^ Cell and Molecular Medicine Research group Shiraz University of Medical Sciences Shiraz Iran; ^5^ Hematology Research Center Shiraz University of Medical Sciences Shiraz Iran; ^6^ Department of Pathology Shiraz University of Medical Sciences Shiraz Iran

**Keywords:** ABC transporter, CD146, dental pulp, drug resistance, fibroblast, stem cell

## Abstract

**Introduction:**

The stem cell portion of the dental pulp derived cultures (DPSCs) showed a higher resistance to cytotoxic effect of restorative dental materials compared to pulpal fibroblasts (DPFs). Here, we aimed to compare the expression of some drug resistant genes between these cells.

**Methods and materials:**

To separate DPSCs from DPFs, we used magnetic cell sorting technique based on CD146 expression. To assess the stem cell properties, the positive and negative portions underwent colony forming assays and were induced to be differentiated into the adipocytes, osteoblasts, hepatocytes, and neural cells. Cell surface antigen panels were checked using immune fluorescence and flow‐cytometry techniques. The mRNA expression of 14 ABC transporters including *ABCA2, ABCB1*, *ABCB11, ABCC1*, *ABCC2*, *ABCC3*, *ABCC4*, *ABCC5*‐2, *ABCC5‐4*,*ABCC*5‐*13, ABCC*6, *ABCC*10, *ABCC11,* and *ABCG2* genes was assessed, using quantitative RT‐PCR technique.

**Results:**

Only the CD146 positive portion could be differentiated into the desired fates, and they formed higher colonies (16.7 ± 3.32 vs. 1.7 ± 1.67, *p* < .001). The cell surface antigen panels were the same, except for CD146 and STRO‐1 markers which were expressed only in the positive portion. Among the ABC transporter genes studied, the positive portion showed a higher expression (approximately two‐fold) of *ABCA2, ABCC5‐13, and ABCC5*‐2 genes.

**Conclusion:**

Dental pulp stem cells which can be separated from dental pulp fibroblasts based on CD146 expression, express higher levels of some drug resistance genes which probably accounts for their features of more resistance to cytotoxic effects of some dental materials. This needs to be more validated in future.

**Abbreviations**
APC: Allophycocyanin
BM: bone marrow
CFU‐F: colony forming unit fibroblast
DPF: dental pulp fibroblast
DPSC: dental pulp stem cell
FITC: fluorescein isothiocyanate
MACS: magnetic activated cell sorting
MNC: mononuclear cells
MSC: mesenchymal stem cell
PE: phycoerythrin
PerCP: peridinin chlorophyll protein
RT‐PCR: Reverse Transcription‐Polymerase Chain Reaction

## 1 INTRODUCTION

Multipotent mesenchymal stromal cells (MSCs) are clonogenic cells with adherence to plastic surfaces and several differentiation abilities (Dominici, Le Blanc, Mueller, 2006). Bone marrow (BM) is known as the common resource of MSCs, whereas other resources, such as adipose tissue (Ahrari et al., 2013), umbilical cord blood (Vasaghi et al., 2013), and dental pulp (Hadaegh et al., 2014) and even some pathological dental tissues such as dental pulp polyps (Attar et al., 2014) and pyogenic granulomas (Dehghani Nazhvani et al., 2018), are also appropriate options. Dental pulp is an ecto‐mesenchyme‐derived tissue, which is created through an early interaction between the mesenchyme and neural crest. Comparative studies of DPSCs and BM‐MSCs revealed many common features, but DPSCs are more specialized and are committed to differentiate into an odontogenic fate instead of osteogenic one and also show a less replicative potential (Huang, Gronthos, & Shi, 2009).

Common DPSCs cultures, like BM‐MSC, are easily accessible via direct culturing of single‐cell suspensions derived from human tissues. They have been found very heterogeneous and are frequently contaminated by the fibroblasts (Huang et al., 2009). Fibroblasts are plastic adherent mesenchymal cells associated with the development, repair, and maintenance of tissues by extracellular matrix production and reforming. Unlike stem cells, they are unable to be differentiated into another cell types (Elkhattouti, Hassan, & Gomez, 2015). Multiple passages for reaching large numbers of DPSCs are needed to fulfill the adequate numbers needed for clinical applications (Vasaghi et al., 2013). However, DPSCs lose their differentiation potentials at later passages (Halfon, Abramov, Grinblat, & Ginis, 2011) mainly by presence of mature cells including the fibroblasts within the adherent fraction of pulpal‐derived mononuclear cells, or because the adherent cells' phenotype changes to that of the fibroblasts over time (Ho, Wagner, & Franke, 2008). Accordingly, recognition and deletion of fibroblasts from these heterogeneous cultures result in improved output of DPSCs as well as their ability for differentiation. Previously, it has been shown that CD146 enrichment can contribute to discrimination of the stem cells from the fibroblasts and cause more resistance to cytotoxic effect of dental composites (Shafiei, Tavangar, Razmkhah, Attar, & Alavi, 2014). Although, compared to DPFs, the mechanism underlying DPSCs resistance to differential cytotoxic agent has not yet been well understood, it may be associated with the expression of drug resistance proteins. Here, we aimed to compare the expression of ABC gene family in DPSC versus DPF as a probable underlying mechanism for the resistance to cytotoxic materials. There are 46 proteins within ABC family. We have evaluated only those with a proven drug resistance activity.

## 2 MATERIAL AND METHODS

### 2.1 Preparing human pulp derived from single‐cell suspensions

Third molars were extracted from 20 to 25‐year‐old adults. The subjects were asked to sign the written informed consent. The local ethics committee approved the search protocol, which was based on the Helsinki declaration. Through a fissure bur, the samples were cut from around the cement‐enamel junction. We removed the pulp tissue mildly from the chambers followed by digestion with an enzyme solution including collagenase type I (3 mg/mL) and 4 mg/mL dispase II (30–60 min/37°C) (Sigma). After centrifugation of the digest (1,200 rpm/5 min), the pellets were suspended.

### 2.2 Cell culture

Single‐cell suspensions were plated in DMEM treated by GlutaMAX (4 mM), penicillin (100 U/mL), streptomycin (100 μg/mL), and 20% FBS (Gibco/Invitrogen). Culturing was performed at 37°C by providing 5% CO2 with humidity level of 90%. We changed the culture media two times a week to obtain the confluence of 80%. In the next step, the obtained cells were released using trypsin–EDTA (Gibco) and subcultured, followed by passaging three times prior to CD146 magnetic cell sorting.

### 2.3 Magnetic‐activated cell sorting (MACS)

For sorting the cells into CD146^+^ cells and CD146^−^ negative cells, MACS was done. Through Dead Cell Removal Kit (Miltenyi Biotec GmbH, Germany), dead cell elimination was done for reducing the risk for nonspecific antibody binding. We labeled the cells via FITC‐conjugated CD146 antibody (BD Biosciences). Anti‐FITC microbeads were used for labeling the target cells (Miltenyi Biotec). MACS was done based on the manufacturer's recommendation. CD146^+^ cells and CD146^−^ were subjected to the experiments for confirming the stemness, such as colony forming assay, differentiations, and cell surface antigens through flow cytometry (as follows).

### 2.4 Colony‐forming unit (CFU) assay

Single cell‐derived colony formation effectiveness was assessed, using CFU seeding unsorted pulp cells and CD146^+^ and CD146^−^ in 6‐well plates (1,000 cells/well). A colony consisting of at least 50 cells was determined. The number of colonies was calculated a day prior to merging the colonies or 14 days after culturing.

### 2.5 Flow cytometry

Analysis of cell‐surface antigen expression levels was performed by incubation of the isolated cells. Antihuman antibodies included CD90–Allophycocyanin (APC), CD34‐Fluorescein isothiocyanate (FITC), CD45‐Peridinin chlorophyll protein (PerCP) (Miltenyi Biotec, Germany), CD14‐FITC, CD166‐Phycoerythrin (PE), CD44‐FITC, CD146‐FITC, HLA‐DR‐PerCP, CD73‐PE (BD Biosciences), and STRO1‐pure (secondary anti‐IgM PE conjugated IgG; Santa Cruz Biotechnology Inc., USA).BD FACSCalibur™ was employed for flow cytometric analysis.

### 2.6 Multi‐lineage differentiation assessment

For adipocyte differentiation of passage 3 cells separated by MACS, culturing of CD146^+^ and CD146^−^ cells was done in the MesenCult medium containing 10% Adipogenic Stimulatory Supplements (Stem Cell Technologies Inc, Canada) based on the instructions. It was approved by analyzing the cells, using Oil‐red O staining and Reverse Transcription‐Polymerase Chain Reaction (RT‐PCR).

In order to osteogenic of passage 3 cells separated by MACS, culturing of the cells was performed in NH‐osteoDiff Medium (Miltenyi Biotec) based on its instruction and was approved by staining cells by Alizarin red as well as RT‐PCR.

For neural differentiation of passage 3 cells separated by MACS, their media was replaced by neurogenic media (neurobasal medium, 2% B27, 20 ng/mL bFGF, 10 μ M retinoic acid) at passage 3–4. The media was changed every 2 days till 14 days.

For differentiation of the cells to hepatocytes of passage 3 cells separated by MACS, cells were serum deprived for 2 days and then were cultured in DMEM supplemented with 10 ng/mL basic fibroblast growth factor (bFGF) (Sigma, USA) and 20 ng/mL epidermal growth factor (EGF) (Sigma, USA). Then a two‐step differentiation protocol was performed as follows: Step‐1 was accomplished by adding DMEM supplemented with 10 ng/mL bFGF, 4.9 mmol/L nicotinamide (Sigma), and 20 ng/mL hepatocyte growth factor (HGF) (Sigma) for 7 days. In step 2, DMEM supplemented with 1 μmol/L dexamethasone (Sigma), 20 ng/mL oncostatin M (OSM) (Sigma), 1.25 mg/mL bovine serum albumin (BSA) (Sigma),10 μL/mL ITS (insulin, transferrin, selenious acid) (Sigma), and 190 μmol/L linoleic acid (Sigma) was added to the cells in order to complete cell maturation up to day 21 and hepatic differentiation was assessed morphologically and by qRT‐PCR method for Thy‐1, albumin (ALB), HNF4α and CYP2E1.

### 2.7 Immune‐fluorescent staining

For evaluation of cell surface marker antigens on the cells separated after MACS, cells were stained with CD146‐FITC (BD Biosciences), CD105‐PE, and CD90‐FITC (Miltenyi Biotec, Germany) based on the protocols for immuofluorescent staining.

To confirm neural differentiation, immunostaining was performed for ß‐tubulin III (Promega cat number: G7121) as a specific antibody against neurons. Briefly, the cells were fixed with 4% PFA in +4°C for 20 min, after that they were permeabilized with 0.01% Triton for 10 min, following by adding blocking solution (5% BSA) for preventing nonspecific binding of antibody. The cells were washed three times with 1X PBS, then they were incubated with primary antibody at concentration of 1:500 for 1 hr in room temperature; after washing primary antibody with 1X PBS, secondary antibody Alexa flour 568 goat antimouse (Abcam cat number #ab175473) was added to the cells and was kept for 45 min in room temperature. The stained cells were observed with Olympus microscope (BX53).

### 2.8 PCR

Total RNA was extracted from the cells using TRIzol Reagent (Invitrogen, Germany) and was used for preparation of cDNA. For reverse transcription of RNAs to the first‐strand cDNA, the Sensiscript Reverse Transcription kit (Qiagen, Germany) was applied.

To determine the adipocyte and osteoblast marker genes expression levels within the induced cells, we conducted RT‐PCR. RT‐PCR was performed for assessing the osteogenic differentiation and finding the osteopontin (forward: 5’‐TTCCAAGTAAGTCCAACGAAAG‐3′; reverse: 5’‐GTGACCAGTTCATCAGAT TCAT‐3′)and col‐1α1 (forward: 5’‐AAGCCGAATTCCTGGTCT‐3′; reverse: 5’‐TCCAACG AGATCGAGATCC‐3’) mRNA. To assess the differentiation into the adipocytes, two markers, *PPAR‐γ2* (forward: 5’‐TTCTCCTAT TGACCCAGAAAGC‐3′; reverse: 5’‐CTCCACTTTGATTGCACTTTGG‐3’)and*aP2* (forward: 5’‐GCCAGGAATTTGACGAAGTC‐3′; reverse: 5’‐TGGTTGATTTTCCATCCCAT‐3′) were detected. Internal control for this study was β‐actin (forward: 5’‐ATCATGTTTGAGACCTTCAA‐3′; 5’‐CATCTCTTGCTCGAAGTCCA‐3′). By using a reaction mixture, the RT‐PCR cycles were done, including reverse and forward primers (Metabion international AG, Germany), dNTP mixture, 10x PCR buffer, MgCl_2_, Taq DNA polymerase (Fermentas, Life Science, EU) as well as distilled water.

To perform a semiquantitative expression analysis of ABC drug resistance gene family, qRT‐PCR was done using ABI step one system with 2 μL cDNA which was amplified in a total volume of 20 μL containing 10 μL of 2X SYBR Green Master Mix (Fermentas, Canada), 7.4 μL DEPC treated water and 0.3 μL of each 10 pmol forward and reverse primers. Thermal cycling was initiated with denaturation at 95°C for 10 min, followed by 50 cycles: denaturation at 95°C for 10 s, annealing and extension at 55°C for 40 s. All data were compared to beta actin housekeeping gene. Sequences of specific primers for qRT‐PCR of all genes are presented in Table S1.

### 2.9 Statistical analysis

The data were analyzed in SPSS software, version 19.0, for windows (IBM) using non parametric Mann–Whitney *U* test. Graph PadPrism 5 (Inc; San Diego CA) was used for graphical presentation of data. In all statistical analyses a *p* value <.05 was considered significant. (Table S1)

## 3 RESULTS

### 3.1 Culture properties

**FIGURE 1:**
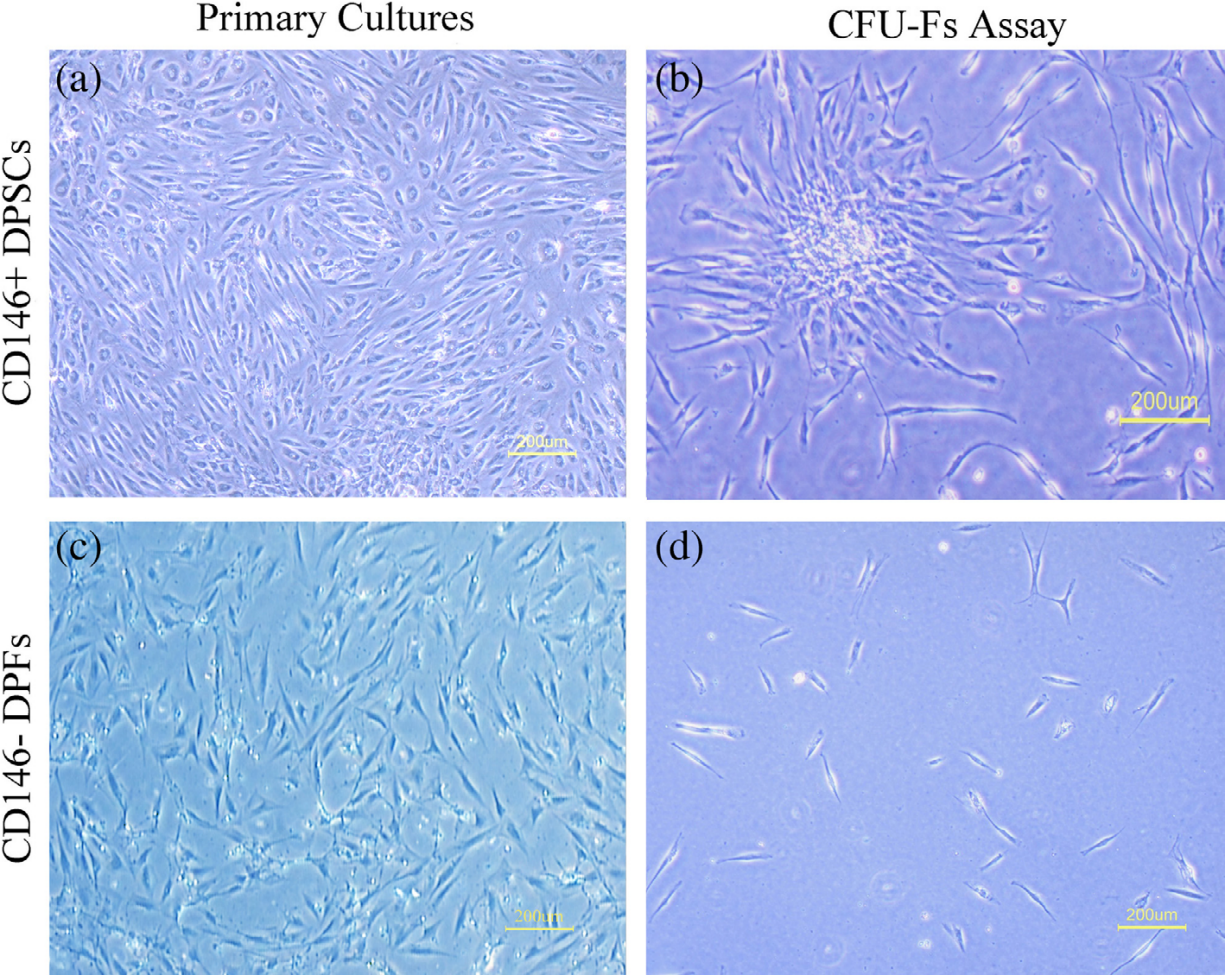
Morphology of cells within cultures: (a) Typical fusiform fibroblast‐like appearance of the cells from CD146 positive; (b**)** A single cell derived colony from CD146 positive derived cells formed within CFU‐Fibroblast assay; (c) CD146 negative cultures; (d) CD146 negative cells could not form any colony. CFU‐F, Colony Forming Unit Fibroblast; DPF, Dental Pulp Fibroblast; DPSC, Dental Pulp Stem Cell

On the second day following the first seeding, attachment of DPCs with the plates and the cultured CD146^+^ and CD146^−^ cells was done. Cell confluence was observed through 12–21 days and the cells showed usual fusiform shape as well as fibroblast‐like morphology (Figure 1).

### 3.2 Clonogenic effect

**FIGURE 2:**
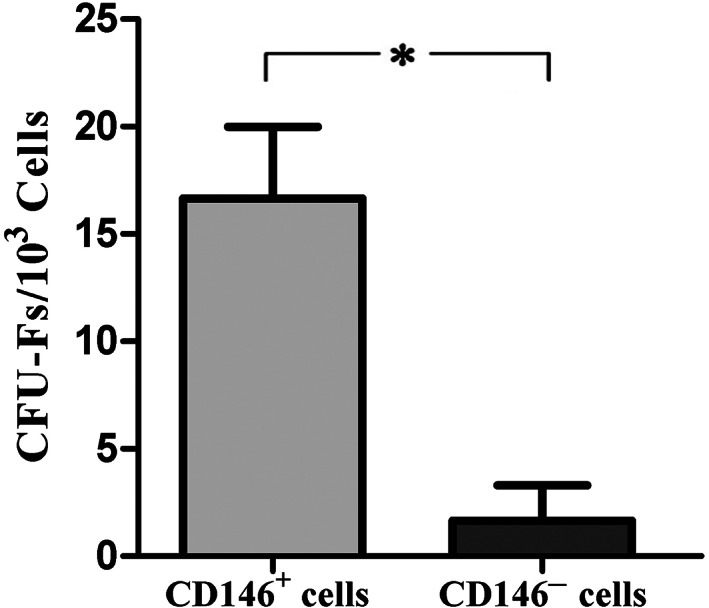
Assessment of Clonogenic efficiency: Initially, 1 × 10^3^ cells were plated in 6‐well culture plates and colony numbers were counted on day 10. Only colonies with more than 50 cells were included in the colony number (*p < .05, n = 4). CFU‐F, Colony Forming Unit Fibroblast

The potential of self‐renewal was analyzed through the CFU assay (n = 4) for determining single cell‐derived colony formation. Each 1,000 CD146 positive cells could form 16.67 ± 3.32 CFU‐Fs (Figure 1b). On the other hand, CD146 negative cells could form 1.7 ± 1.67 colonies (Figure 1d), which was significant (*p* < .001; Figure 2).

### 3.3 Flow‐cytometry outcomes

**FIGURE 3:**
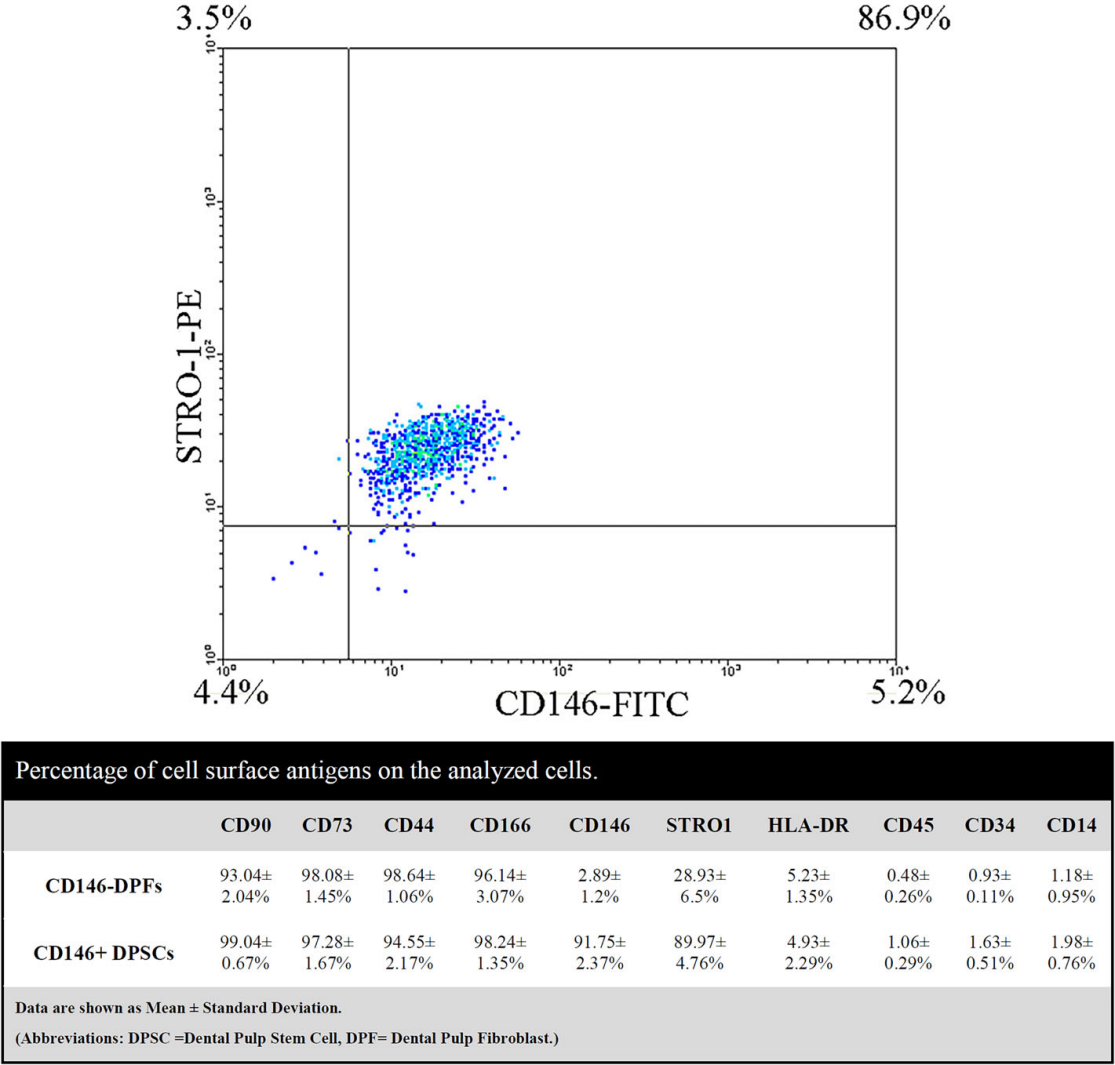
Flow cytometric analysis of the cells: Flow cytometry revealed highly pure isolation of CD146 positive cells in most of the coexpress STRO‐1 marker. Comparison of cell surface antigen marker panel between the CD146 positive and negative portions is provided in the Table

Following CD146 cell sorting, the purity of 91.75 ± 2.37% was obtained for the isolation. CD146^+^ and CD146^−^ cells flow cytometric analysis results indicated that they were positive for mesenchymal markers, like CD44, CD166, CD90, and CD73, and negative for surface molecules, including CD14, CD34, CD45, and HLA‐DR. A uniform positivity was found for STRO‐1 in CD146^+^ portion (89.97 ± 4.76%); however, CD146^+^ portion displayed STRO‐1 expression relatively (28.93 ± 6.5%). Figure 3 illustrates the overall findings of flow cytometry assay.

### 3.4 Immune‐fluorescent staining

**FIGURE 4:**
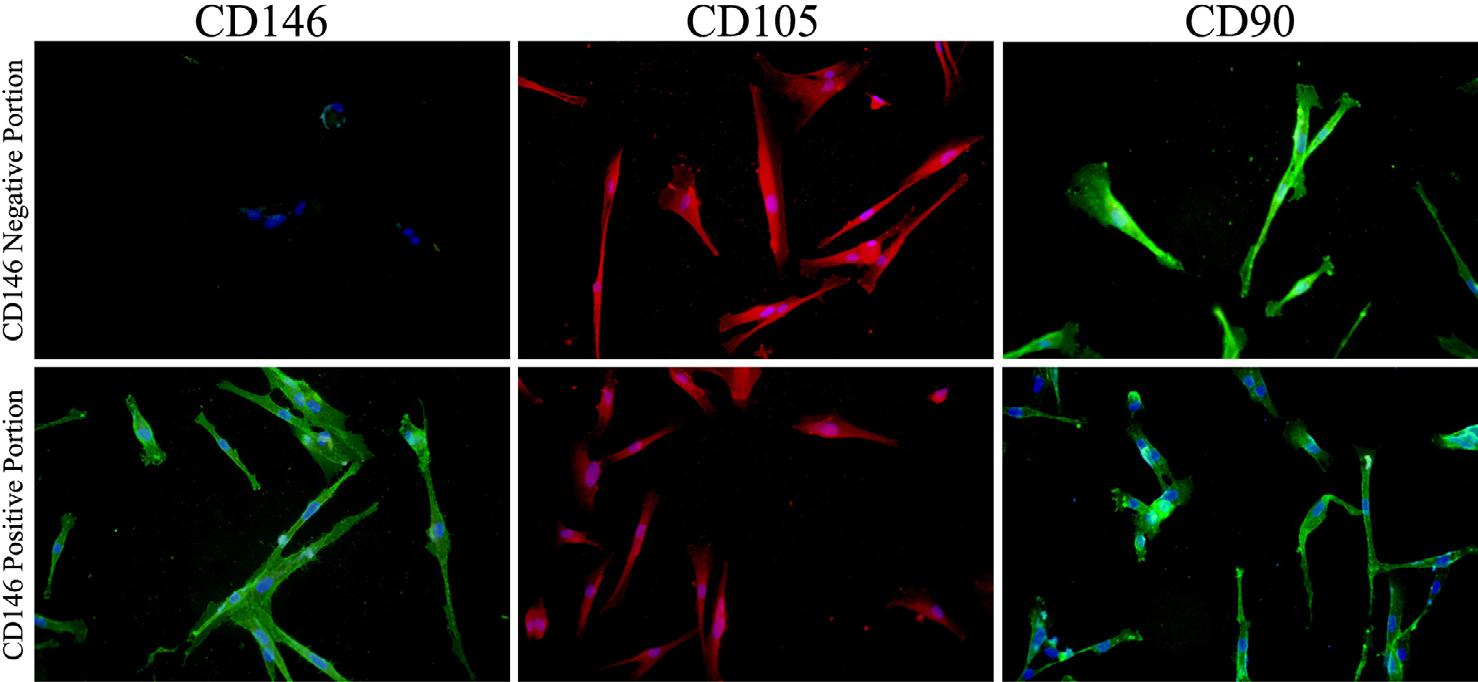
Immunofluoroscent staining: Both the CD146 positive and negative isolated cells showed expression of CD90 and CD105, while they were different in the expression of CD146

As shown in Figure 4, both the CD146 positive and negative isolated cells showed expression of CD90 and CD105 while were different in the expression of CD146.

### 3.5 Differentiation assay

**FIGURE 5:**
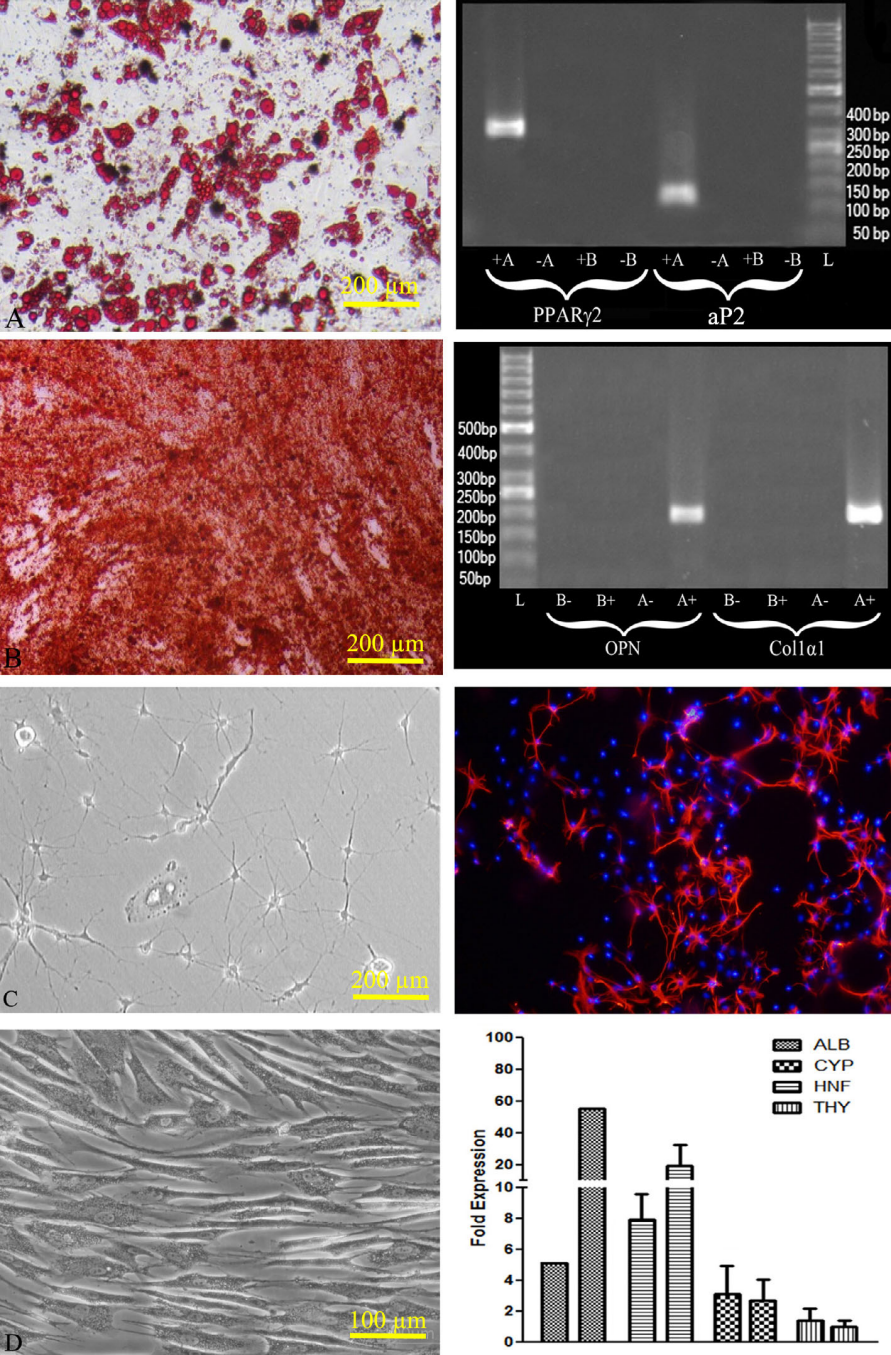
Differentiation assays. (a) Left: Adipocyte with lipid vacuole resulted from adipogenic differentiation of CD146 positive cell stained with oil red. Right: Expression of PAPR‐γ2 and aP‐2 is shown following adipogenic differentiation using PCR. (b) Left: Mineralization and appropriate morphological changes are shown following osteogenic differentiation stained with alizarin red. Right: With osteogenic differentiation, expression of OPN and Col1α1 is revealed by PCR. (c) Left: With neurogenic differentiation typical dendritic cells which express appeared Right: ß‐tubulin III revealed by immune‐fluorescent staining. (d) Left: With hepatocytic differentiation, polygonal/flattened shape cells appeared at day 21 (differentiation step 2) Right: Hepatogenic differentiation was confirmed by qRT‐PCR as hepatogenic related genes were upregulated postdifferentiation, specially ALB and HNF with approximately 10‐ and 2.5‐fold higher expression after differentiation. The bars represent gene expressions before and after differentiation

For confirming multi‐potentiality of the cells, differentiation assay was done. Morphological alterations, like vacuoles production, were considered for confirmation of CD146^+^ cells differentiation to adipocytes (using Oil‐red O staining). *PPAR‐γ2* and *aP2* mRNAs expression based on RT‐PCR approved this differentiation (Figure 5a).

Mineralization, as a marker of osteoblastic differentiation, was evidenced via alizarin red staining. Osteopontin and col‐1α1 based on RT‐PCR results were expressed by differentiated cells (Figure 5b). None of the above changes could be demonstrated in the CD146 negative cells.

Immunostaining showed that CD146 positive cells were differentiated to neurons after exposure to neurogenic media, also it seems that we may have some neural network between the differentiated cells which expressed ß‐tubulin III (Figure 5c).

CD146 positive cells which underwent hepatogenic differentiation, showed morphological changes into polygonal and flattened shapes. Cells showed a fibroblast‐like morphology before differentiation and no significant change was observed at day 7 while a polygonal/flattened shape was appeared at day 21 (differentiation step 2). Hepatogenic differentiation was confirmed by qRT‐PCR as hepatogenic related genes were upregulated post differentiation specially ALB and HNF with approximately 10‐ and 2.5‐fold higher expression after differentiation (Figure 5d).

### 3.6 Expression pattern of drug resistance genes

In the present study the mRNA expression of 14 ABC transporters including*ABCA2*, *ABCB1*, *ABCB11*, *ABCC1*, *ABCC2*, *ABCC3*, *ABCC4*, *ABCC5*‐2, *ABCC5‐4*, *ABCC*5‐13, *ABCC*6, *ABCC*10, *ABCC11*, and *ABCG2* genes were assessed in both CD146 positive and negative cells. All genes showed mRNA expression in both cells except for *ABCB11* and *ABCC11* genes.

**FIGURE 6:**
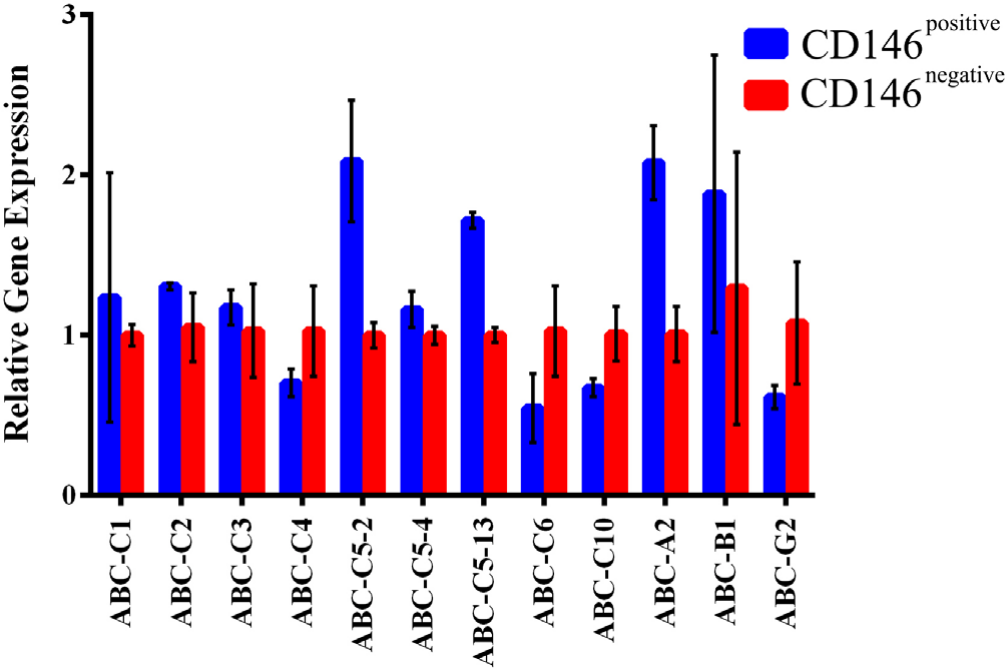
Quantitative real‐time PCR of ABC gene family expression. As it is shown, CD146 positive portion expressesABCA2, ABCC5‐2, and ABCC5‐13 genes in a higher level (n = 4)

Then we compared the expression of ABC transporters between CD146^+^ and CD146^−^ cells. Although no significant difference was found, *ABCA2, ABCC5*‐2, and*ABCC*5‐13 showed approximately two‐fold higher expression in CD146^+^cells whereas *ABCC*6, *ABCC*10, and *ABCG2* genes showed lower expression in CD146^+^ cells compared to the CD146^−^ cells (Figure 6).

## 4 DISCUSSION

In the present study, CD146 enrichment approach was employed for separation of the stem cells from contaminating the fibroblasts from a pulp tissue‐derived routine culture. Based on the results, colonies were only formed by the CD146^+^ portion of the cells and they showed multi‐lineage differentiation capacity, whereas the CD146^−^ portion did not. Consequently, CD146 positive cells seem to be true stem cells (DPSC) and the negative portion is likely to be fibroblasts (DPF). DPSCs showed the differential expression level of ABC gene family, especially *ABCA2, ABCC5‐13, andABCC5*‐2 genes, which may help explain their further resistance to cytotoxic effects of restorative dental materials.

The routine cultures of BM‐MSC (Phinney, 2007) as well as DPSCs (Huang et al., 2009) directly obtained via culturing tissue‐derived single cell suspensions have been found so heterogeneous, which is supported by contaminated stem cells by mature cells, including fibroblasts (Ratajczak, Kucia, Majka, Reca, & Ratajczak, 2004). Based on the current findings, several factors, such as plastic‐adherent and spindle‐shaped cells as well as cell surface markers, like CD14, CD34, CD44, CD45, CD73, and CD105 applied to define the stem cells (Dominici et al., 2006), are not able to differentiate these cells from fibroblasts (Alt et al., 2011). Several studies have been conducted on discriminating factors. It has been indicated that colony‐forming feature can be used to distinguish the stem cells from the fibroblasts (Alt et al., 2011). In another study, Halfon et al. compared a wide panel of cell surface markers, showing that only CD146 expression was restricted to MSCs (Elkhattouti et al., 2015). Furthermore, Zannettino et al. showed that the entire CFU‐Fs from adipose tissue derived MSCs are within the CD146 positive portion (Zannettino, Paton, Arthur, 2008). Such results recommend that CDl46 expression may differentiate the real mesenchymal stem cells from the fibroblasts. Based on that we used CD146 enrichment approach to reach final population of two distinct cell types; DPSCs and DPFs which were needed for the other steps our study. Based on the findings that just CD146^+^ positive portion was able to produce colonies and had a multi lineage differentiation potential; we used CD146 positive cells as DPSCs and CD146 negative cells as DPFs for the other steps of our experiment.

Previously, we have shown that DPSCs are more resistant to the cytotoxic effect of dental composites compared to DPFs (Shafiei et al., 2014). There is limited information on the resistance of mesenchymal stem cells, especially DPSCs, to cytotoxic subjects. Based on the Sauer's results, detectable levels of ATP7B protein can be expressed by BM‐MSCs inhibiting from toxic copper (Erdei, Lorincz, Szebenyi, 2014). Hematopoietic stem cells (HSCs) have been largely examined for resistance to cytotoxic materials. In the hematopoietic hierarchy, the resistance of cells to cytotoxic material reduces by decreasing the stemness of the cells and appearance of more mature phenotype, which can be due to the expression of proteins belonging to the ATP‐binding cassette (ABC) transporters. Their high expression can protect the cells from cytotoxic medications via an ATP‐dependent drug efflux technique. There is a correlation between an undifferentiated condition in normal HSCs and ABCB1and ABCG2 belonging to the ABC transporter genes. ABCG1, ABCA1, ABCB1, ABCC1, ABCD4, and ABCB2 genes expression is mostly seen in hematopoietic stem cells and their down‐regulation can be observed after differentiation into a progenitor state (Aberuyi et al., 2017). ABC multidrug transporters have crucial roles in cellular homeostasis and provide defense mechanism against toxic agents. Recently, there has been heightened interest in the differential expression of these genes in different types of stem cells and differentiated and undifferentiated cell types. There are reports demonstratingABCG2, ABCA1, and ABCB6 expression in human embryonic stem cell lines. High expression level for ABCB6 and ABCC1 in the embryonic stem cell derived neural cells and high expression of ABCB6 was observed in the human embryonic‐stem‐cell‐derived cardiomyocytes (hESC‐CMs). In the mesenchymal‐like stem cells, ABCA1, ABCB6, and ABCC1 were detected, whereas no expression for ABCB1 or ABCG2 was reported (Erdei et al., 2014). In the present study, the mRNA expression of 14 ABC transporters was examined in the CD146^+^dental pulp derived stem cells and CD146^−^ cells. Based on the results, ABCA2, ABCC5‐13, and ABCC5‐2 were predominantly expressed in the CD146^+^ stem cells compared to the other genes and CD146^−^ cells. ABCA2 is highly expressed in brain tissue and may play a role in macrophage lipid metabolism and neural development. It is shown that the protein level of ABCA2 has a prognostic impact on pediatric ALL MDR (Aberuyi et al., 2017). ABCC5, also known as MRP5 (multidrug resistance‐associated protein 5), is a member of the C branch of the superfamily of ATP‐binding cassette transporters, which use the energy provided by the hydrolysis of ATP to transport substrates across the plasma membrane. In terms of drug resistance, ABCC5 is best known for its ability to transport nucleotide analogs, antifolates like methotrexate, cyclic nucleotides, folic acid, and the recently identified *N*‐lactoyl‐amino acids (Jansen, Mahakena, de Haas, Borst, & van de Wetering, 2015). The precise role of these genes in DPSCs needs further investigation.

Altogether, CD146 marker can be used for separating DPSCs from DPF in the cultures of the pulp‐derived cells. DPSCs express some drug resistance genes including ABCA2, ABCC5‐2, and ABCC5‐13, as compared to DPF. Whether these genes caused DPSC to be more resistant to cytotoxic effects of dental composites compared to DPFs is a matter of future investigations.

## DISCLOSURE OF INTEREST

The authors have no commercial, proprietary, or financial interest in the products or companies described in this article.

## AUTHORS' CONTRIBUTION

Conception: Armin Attar, Mahboobeh Razmkhah; Design: Maryam S. Tavangar, Ahmad Monabati; data acquisition: Maryam S. Tavangar, Mahboobeh Razmkhah, Seyed‐Mojtaba Hosseini, Ahmad Hosseini; Data analysis: Armin Attar, Fereshteh Shafiei; data interpretation: Fereshteh Shafiei; Drafting: Maryam S. Tavangar, Armin Attar, Mahboobeh Razmkhah; Critical revision: Maryam S. Tavangar, Armin Attar, Mahboobeh Razmkhah, Seyed‐Mojtaba Hosseini, Ahmad Hosseini, Ahmad Monabati, Fereshteh Shafiei; Final approval: Maryam S. Tavangar, Armin Attar, Mahboobeh Razmkhah, Seyed‐Mojtaba Hosseini, Ahmad Hosseini, Ahmad Monabati, Fereshteh Shafiei;

## Supporting information


**Table S1** The sequences of primers used for detecting the expression of different ABC transporter genes.
