# Supplementary material for: Differential expression of drug resistance genes in CD146 positive dental pulp derived stem cells and CD146 negative fibroblasts
Source: Clin Exp Dent Res. 2020 May 7;6(4):448–56. doi: 10.1002/cre2.297 (PMC7453779; doi:10.1002/cre2.297)
Supplement: Supplementary file 1 — Table S1 The sequences of primers used for detecting the expression of different ABC transporter genes. [file CRE2-6-448-s001.docx]

**S.Table 1.** The sequences of primers used for detecting the expression of different ABC transporter genes

| Primer name | Primer Sequence |
| --- | --- |
| ABC-G2 Forward | CGTGGAACTCTTTGTGGTAG |
| ABC-G2 Reverse | CCTTTGGCTTCAATCCTAACA |
| ABC-C6 Forward | CGTGGTCTGCTTCGTCTA |
| ABC-C6 Reverse | TTGCTCCTCCTGATGGTG |
| ABC-A2 Forward | GCCGTCCTCTCCCTCTTC |
| ABC-A2 Reverse | TCAGGTCCTTGTCGTGCTC |
| ABC-B11 Forward | TTACTGGATTCGTGTGGTGTCT |
| ABC-B11 Reverse | TGATGGGTTTCCTGTCTATTGTCT |
| ABC-B1 Forward | CCATGCTCAGACAGGATGTGA |
| ABC-B1 Reverse | AGTTGCCAACCATAGATGAAGGA |
| ABC-C1 Forward | ACCATCCACGACCCTAATCC |
| ABC-C1 Reverse | CGCATTCCTTCTTCCAGTTCTT |
| ABC-C2 Forward | GGACACTCTTACAGGGTGACAA |
| ABC-C2 Reverse | AGGAATGAAGCTATGGATGATGGA |
| ABC-C3 Forward | CCAAGGCAGAGGGTGAGAT |
| ABC-C3 Reverse | ACAGGCGGGAGAGAAAGC |
| ABC-C4 Forward | GTGTTCTTCTGGTGGCTCAATC |
| ABC-C4 Reverse | AGGCTTCTGTGCGTCATTCT |
| ABC-C5-2 Forward | GCGAAGGGTTGTGTGGAT |
| ABC-C5-2 Reverse | AAGGAAGGCTGAACTCTTGG |
| ABC-C5-13 Forward | GCTCCGCCACTGTAAGATTCT |
| ABC-C5-13 Reverse | TGTCAAACTCCACCACCTGTC |
| ABC-C5-4 Forward | CACTTGCTTGGTGCTGTCA |
| ABC-C5-4 Reverse | AGGAAGGCTGAACTCTTGGC |
| ABC-C10 Forward | CGGTGCTGCTGATGGAGG |
| ABC-C10 Reverse | CTGGGCTGTGGCTGAGTC |
| ABC-C11 Forward | GGGCAATGGGAAGGTGGTA |
| ABC-C11 Reverse | AACAAGAAGGTCGCAGACTGT |
